# Tryptophan metabolism-related gene expression patterns: unveiling prognostic insights and immune landscapes in uveal melanoma

**DOI:** 10.18632/aging.205122

**Published:** 2023-10-13

**Authors:** Jing Wang, Tienan Zhao, Bo Li, Wei Wei

**Affiliations:** 1 Department of Ophthalmology, Shengjing Hospital of China Medical University, Shenyang, Liaoning, China; 2 Institute of Biological Sciences, Faculty of Science, University of Malaya, Kuala Lumpur, Malaysia; 3 College of Network and Continuing Education, China Medical University, Shenyang, Liaoning, China

**Keywords:** tryptophan, uveal melanoma, metabolism, immune infiltration, tumor microenvironment

## Abstract

Uveal melanoma (UVM) remains the leading intraocular malignancy in adults, with a poor prognosis for those with metastatic disease. Tryptophan metabolism plays a pivotal role in influencing cancerous properties and modifying the tumor’s immune microenvironment. In this study, we explore the relationship between tryptophan metabolism-related gene (TRMG) expression and the various features of UVM, including prognosis and tumor microenvironment. Our analysis included 143 patient samples sourced from public databases. Using K-means clustering, we categorized UVM patients into two distinct clusters. Further, we developed a prognostic model based on five essential genes, effectively distinguishing between low-risk and high-risk patients. This distinction underscores the importance of TRMGs in UVM prognostication. Combining TRMG data with gender to create nomograms demonstrated exceptional accuracy in predicting UVM patient outcomes. Moreover, our analysis reveals correlations between risk assessments and immune cell infiltrations. Notably, the low-risk group displayed a heightened potential response to immune checkpoint inhibitors. In conclusion, our findings underscore the dynamic relationship between TRMG expression and various UVM characteristics, presenting a novel prognostic framework centered on TRMGs. The deep connection between TRMGs and UVM’s tumor immune microenvironment emphasizes the crucial role of tryptophan metabolism in shaping the immune landscape. Such understanding paves the way for designing targeted immunotherapy strategies for UVM patients.

## INTRODUCTION

Uveal melanoma (UVM), also termed ocular melanoma, is a unique neoplasm that arises from malignancies within the uvea [1]. It affects approximately 5–7 individuals per million each year, with management typically involving surgical procedures, brachytherapy, or proton beam therapy [2–4]. Genetic anomalies play a critical role in UVM development and progression [5, 6]. Key genetic players, such as SF3B1 [6], PTP1B [7], BAP1 [8], and EIF1AX [9], serve both as indicators of prognosis and as therapeutic targets. While localized UVM cases have a promising 5-year survival rate of around 80%, metastatic instances often exhibit a dire prognosis, with an average survival duration of less than one year [10]. Thus, understanding prognosis-related biomarkers or risk factors for UVM is vital for enhancing clinical intervention strategies.

Tryptophan (Trp) is an essential amino acid that must be obtained from dietary sources due to its absence of endogenous synthesis [11]. Its metabolic pathway primarily branches into the serotonin and kynurenine pathways (KP) [12]. Key rate-limiting enzymes identified within the KP include TDO, IDO1, and IDO2 [13, 14]. The multifaceted roles of these enzymes in various diseases – from cancers [15] to autoimmune conditions [16] and neurological disorders [17] – make them compelling subjects for scientific exploration. Previous studies have demonstrated that TDO’s presence in glioblastoma [18] and breast cancer [19] cells can enhance malignancy. Similarly, overexpression of IDO1 increases the mobility of lung cancer cells, whereas its inhibition reverses this effect [20]. Additionally, in cervical cancer-related lymph nodes, a significant proximity was observed between FOXP3+ Treg cells and dendritic cells expressing IDO1 [21]. This interaction correlates with elevated levels of CD4+CD25+FOXP3+ Treg cells in patients with conditions like AML [22] and metastatic pancreatic ductal adenocarcinoma [23]. Recent findings also indicate that tumor cells can transfer Kyn to CD8+ T cells, prompting an increase in PD-1 expression through an AHR-dependent process [24]. Furthermore, TDO displays substantial immunosuppressive capabilities, inhibiting T-cell growth and reducing immune cell infiltration into the tumor environment [25]. These studies underscore Trp’s central role in tumor progression and immune response modulation [26]. While tryptophan metabolism (TPM) profoundly impacts the immune aspects of tumors, its influence on UVM’s progression and immune landscape remains an area ripe for exploration.

In our recent work, we studied TCGA and GEO cohorts of UVM patients. Our aim was to discern the relationship between TRMGs expression and the immunological and morphological traits of UVM. We conducted extensive analyses to decipher the intricate dynamics between TRMGs and UVM’s immune status. Additionally, we assessed drug sensitivity in UVM patients based on TRMG profiles. By integrating our findings with current knowledge, we are poised to delve deeper into how TPM might augment responses to immune checkpoint inhibitors and guide immunotherapy choices for UVM patients.

## METHODS

### Study population and data collection

UVM mRNA expression profiles were explored in a cohort of 80 patients, with data sourced from The Cancer Genome Atlas (TCGA) database (https://portal.gdc.cancer.gov/) and the Gene Expression Omnibus (GEO, GSE22138). Throughout this research, strict adherence to relevant guidelines ensured methodological rigor. Duplicates were removed, and cases lacking clinical outcomes were excluded (processed datasets were shown in Supplementary File 1). For data processing, the R programming language was employed, effectively translating Ensembl ID numbers into gene symbols.

### Defining tryptophan metabolism-related genes

In our exploration of TMRGs (Supplementary File 2), we focused on those associated with the “KEGG TRYPTOPHAN METABOLISM” pathway, drawing data from The Molecular Signatures Database (MSigDB) (http://software.broadinstitute.org/gsea/msigdb/index.jsp).

### Alterations of TMRGs in UVM

TMRG modifications in the TCGA database, which include changes in gene expression, methylation, and copy number variations (CNVs), were analyzed using the Gene Set Cancer Analysis platform (http://bioinfo.life.hust.edu.cn/GSCA/#/). Our study assessed the impact of these TMRG alterations on disease-free survival (DSS), overall survival (OS), and progression-free survival (PFS) in patients.

### Identification of subtypes and biological function enrichment

Using consensus unsupervised clustering analysis, patients were classified into specific molecular subgroups based on the expression patterns of 40 TRMGs. We then assessed the differences in prognosis and survival rates among these subtypes, employing univariate Cox regression and Kaplan-Meier survival analyses with the aid of the ‘survival’ (3.5.7) and ‘survminer’ (0.4.9) R packages. Additionally, using the Gene set variation analysis (GSVA), we determined the variations in molecular subtype enrichment within biological processes, referencing the Kyoto Encyclopedia of Genes and Genomes (KEGG) and Gene Ontology (GO) gene sets.

### Establishment of the risk model

To construct a precise prognostic signature focusing on minimal RNA processing factors, we applied the least absolute shrinkage and selection operator (LASSO) penalty using R software’s “glmnet” (4.1.8) package. Ten-fold cross-validation determined the optimal penalty parameter (λ) and established regression coefficients for individual variables. The formula used was RiskScore = Σβi × Expi, where i represents gene expression levels and β corresponds to the gene’s Cox regression coefficient. Hazard ratios emerged from both univariate and multivariate Cox regression analyses, visualized through forest plots generated by R’s “forestplot” (3.1.3) package.

### Evaluation of the TRMGs prognostic model

Based on the median risk score, patients were classified into either high- or low-risk group. The efficacy of the model was assessed by comparing survival rates between these groups using Kaplan-Meier plots generated via R’s “survival” package. Differences in CRG expression between the groups were illustrated using a heatmap created with the “pheatmap” package. Time-dependent receiver ROC analysis, facilitated by the “survminer” and “timeROC” R packages, assessed the model’s specificity and sensitivity over 1-, 3-, and 5-year periods. The area under the ROC curve (AUC) indicated the model’s predictive accuracy. A nomogram for predicting UVM patient survival over 1-, 3-, and 5- year durations was formulated using the “rms” package, integrating factors like age, pathologic stage, T-stage, gender, and the risk score.

### Analysis of immune capacity and drug susceptibility across risk categories

We employed CIBERSORTx to calculate the tumor infiltrating immune cells (Supplementary File 3), and then evaluate correlations with risk scores. The research also identified differential expressions of immune cell subsets between the high- and low-risk groups. To explore variations in drug sensitivity between the risk groups, we utilized the ‘pRRophetic’ (0.5) algorithm and ‘ggpubr’ (0.6.0) packages, computing IC50 values for commonly used immunotherapeutic drugs in cancer treatment.

### Immune cell infiltration assessment

The gene markers were retrieved from Immune Cell Abundance Identifier (ImmuCellAI), which was shown in Supplementary File 4. We compared immune cell infiltration patterns across 24 immune cells (ImmuCellAI) between high- and low-risk groups. Grouping 143 UVM patients based on median risk scores, we analyzed data on the platform to understand the dynamics of immune cell abundance and infiltration.

### Cell culture

UVM cell lines C918 and M619, sourced from ATCC (VA, USA), were cultivated in DMEM supplemented with 10% fetal bovine serum and incubated at 37°C with 5% CO2. Post 24-hour treatment with 5 μM N,N-Dimethyltryptamine (DMT), one group was established as the DMT group, while the other served as a control (NC group).

### Cell viability assay

5 × 10^3^ cells were seeded onto a 96-well plate and allowed to adhere overnight in a medium containing 10% FBS. After the initial treatment, cell viability was gauged using the Cell Counting Kit-8. Post-DMT treatment, the cells were left to form colonies for seven days. Following this period, the cells were fixed, stained, and colony numbers were quantified using ImageJ software.

### Colony formation assay

Following DMF treatment, cells were seeded in 6-well plates at a density of 2 × 10^3^ cells/well and incubated in DMEM supplemented with 20% FBS for seven days. After this period, cells were fixed using 70% ethanol and stained with a 0.25% crystal violet solution (Thermo-Fisher, PA, USA). Colonies were then quantified using ImageJ software.

### Cell apoptosis

Using flow cytometry, apoptosis was analyzed with the Annexin V-FITC cell apoptosis detection kit (Beyotime, Shanghai, China). Detached cells were suspended in 1× binding buffer at a concentration of 1 × 10^5^ cells/mL. Following this, 5 μL of FITC Annexin-V and 5 μL of propidium iodide were added to 100 μL of the cell suspension, which was then incubated in the dark for 15 minutes. After incubation, 400 μL of 1× binding buffer was added. Apoptosis was analyzed using the FACS Calibur flow cytometer with Cell-Quest software (BD Cell Quest Pro Software, BD Biosciences, CA, USA).

### Cell invasion assay

Transwell cell culture systems with 8 μM pores in the upper chamber membranes were used for cell invasion assays. The upper chamber membrane was coated with a mixture of Matrigel (Corning, NY, USA) and serum- free DMEM. 1 × 10^4^ cells in suspension were seeded into each upper chamber in serum-free medium, with or without 5 μM DTM. The lower chamber was filled with DMEM medium containing 20% FBS. After a 24-hour incubation at 37°C and 5% CO2, the cells were fixed with 4% formaldehyde and stained with 0.1% crystal violet. Non-invaded cells on the upper membrane were removed using a 70% ethanol-soaked cotton swab. Invaded cells on the lower side of the membrane were visualized and captured with an inverted microscope (Ti-S, Nikon, Tokyo, Japan). Using ImageJ software, the invaded cells were counted, and an average value was calculated from three randomly chosen fields of view, each at 100× magnification.

### Scratch wound-healing migration assay

UVM cells were cultured on a six-well plate at a density of 5 × 10^5^ cells per well and incubated until they reached confluence. Using a 10 μL pipette tip, a linear scratch was made across the monolayer. The cells were then treated with either 0 or 12.5 nm of DMT. Images were captured at 0 and 24-hour intervals using an inverted light microscope (Leica DM IL, Wetzlar, Germany). The wound area was analyzed with ImageJ software, and the wound closure rate was calculated using the formula: (initial wound area - residual wound area)/initial wound area ×100%.

### Statistical analysis

For comparisons between two groups, the Student’s *t*-test was employed. For multiple group comparisons, the Wilcoxon test was used. Survival differences were assessed using the log-rank test. A *p*-value of less than 0.05 was deemed statistically significant. The code script was shown in Supplementary File 5.

### Data availability statement

The datasets presented in this study can be found in online repositories.

## RESULTS

### The difference in survival according to alteration of TMRGs in patients with UVM

**Figure 1:**
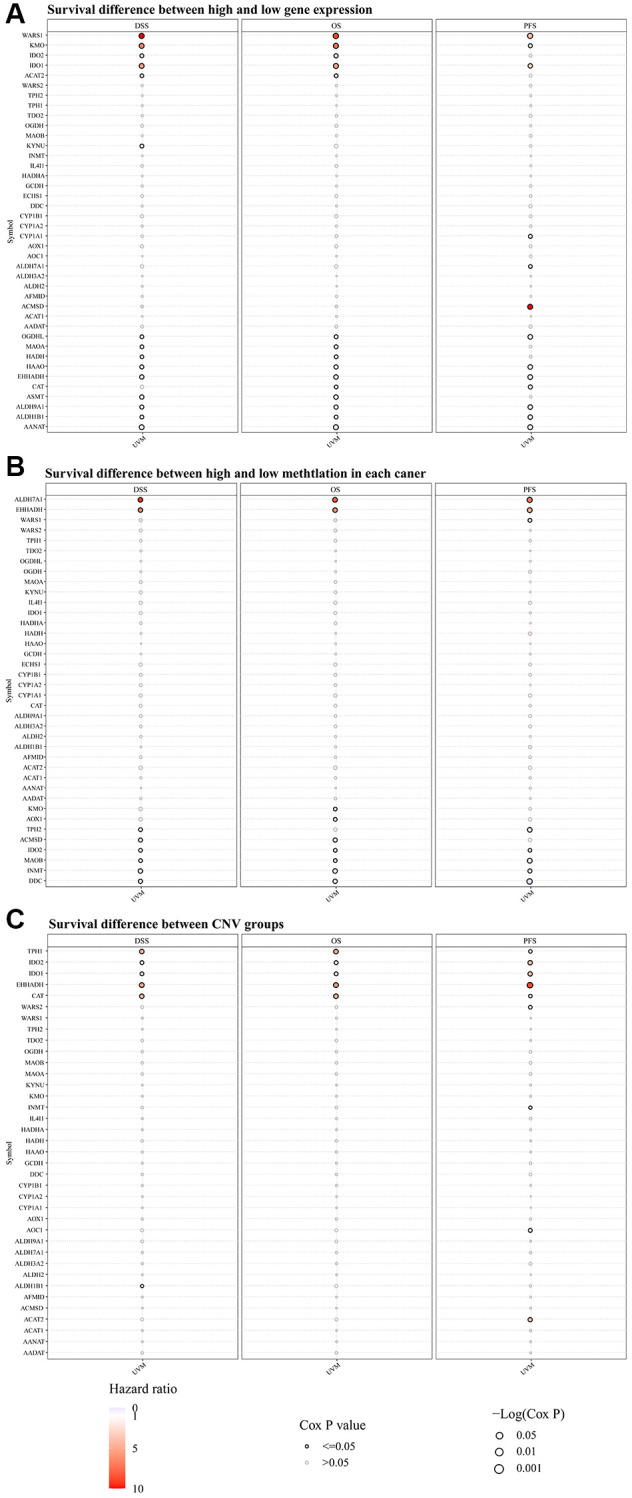
**The disparity in survival outcomes in the modifications of TMRGs among patients afflicted with UVM.** (**A**) The distinction in survival outcomes between elevated and diminished TMRG expression profiles; (**B**) Survival difference between high and low methylation of TMRGs; (**C**) Survival difference between CNV groups of TMRGs. TMRGs, tryptophan metabolism-related genes. The color from blue to red represents the hazard ratio, size represents statistical significance in bubble plot. The black outline border indicates Cox *P* ≤ 0.05.

In assessing the survival differences linked to TMRGs alterations in UVM patients, a thorough analysis of the mRNA expression of TMRGs in the UVM cohort from TCGA revealed a significant correlation. High expression levels of WARS1, KMO, and IDO1 were associated with shorter DSS, OS, and PFS. Conversely, low expression of OGDHL, HAAO, EHHADH, ALDH9A1, ALDH1B1, and AANAT yielded similar outcomes (Figure 1A). Additionally, an examination of TMRGs methylation levels classified UVM patients into two groups: those with hazard ratios exceeding 1 and those below. It was found that higher methylation of ALDH7A1 and EHHADH, and lower methylation of IDO2, MAOB, INMT, and DDC were indicative of unfavorable DSS, OS, and PFS outcomes (Figure 1B). A subsequent survival analysis using CNV data in UVM confirmed that CNV of TPH1, IDO2, IDO1, EHHADH, and CAT were also associated with poor DSS, OS, and PFS (Figure 1C).

### Establishment of Trp subtypes and analysis of biological pathways

**Figure 2:**
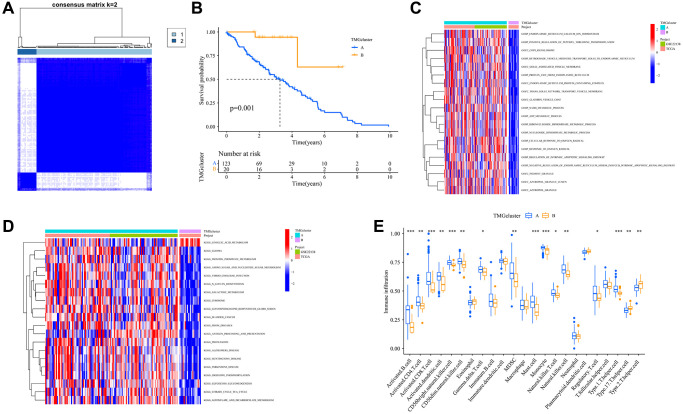
**Trp-related dual classifications and the enrichment of pertinent biological functions.** (**A**) Harmonized matrix heatmap delineating a pair of distinct clusters; (**B**) Survival analysis among the two subtypes; *P* value was calculated based on Log-rank test; (**C**) Differences in the PCA analysis among the two clusters; (**D**) Disparities in expression levels of TMRGs related to clinical characteristics within the two unique subtypes; (**E**) Disparities in the immune infiltration between the two distinct clusters. Abbreviations: Trp: tryptophan metabolism; KEGG: Kyoto Encyclopedia of Genes and Genomes; GO: Gene Ontology.

To elucidate the role of TMRGs in UVM progression, we analyzed various genomic factors. We classified patients from TCGA and GSE22138 into two distinct clusters based on the consensus matrix (Figure 2A). Notably, cluster A exhibited a significantly prolonged OS compared to cluster B (Figure 2B). A GSVA in GO revealed enrichment in metabolic processes (such as NADH, ADP, ribonucleoside diphosphate, and nucleoside diphosphate) as well as vesicle and granule pathways in cluster A (Figure 2C). Similarly, a GSVA in KEGG showed that cluster A was rich in diverse pathways, including cancer (glioma and bladder cancer) and metabolic pathways (like linoleic acid, glyoxylate and dicarboxylate, inositol phosphate, and galactose) (Figure 2D). Delving deeper with ssGSEA, we observed greater immune cell infiltration in cluster A, characterized by the prevalence of immune cell types such as Activated. B.cell, Activated (Figure 2E). CD4.T.cell, Activated.CD8.T.cell, and others. Our findings suggest that TMRGs may play a pivotal role in UVM progression, potentially by regulating immune-related and metabolic processes and pathways.

### Identification of gene subtypes

**Figure 3:**
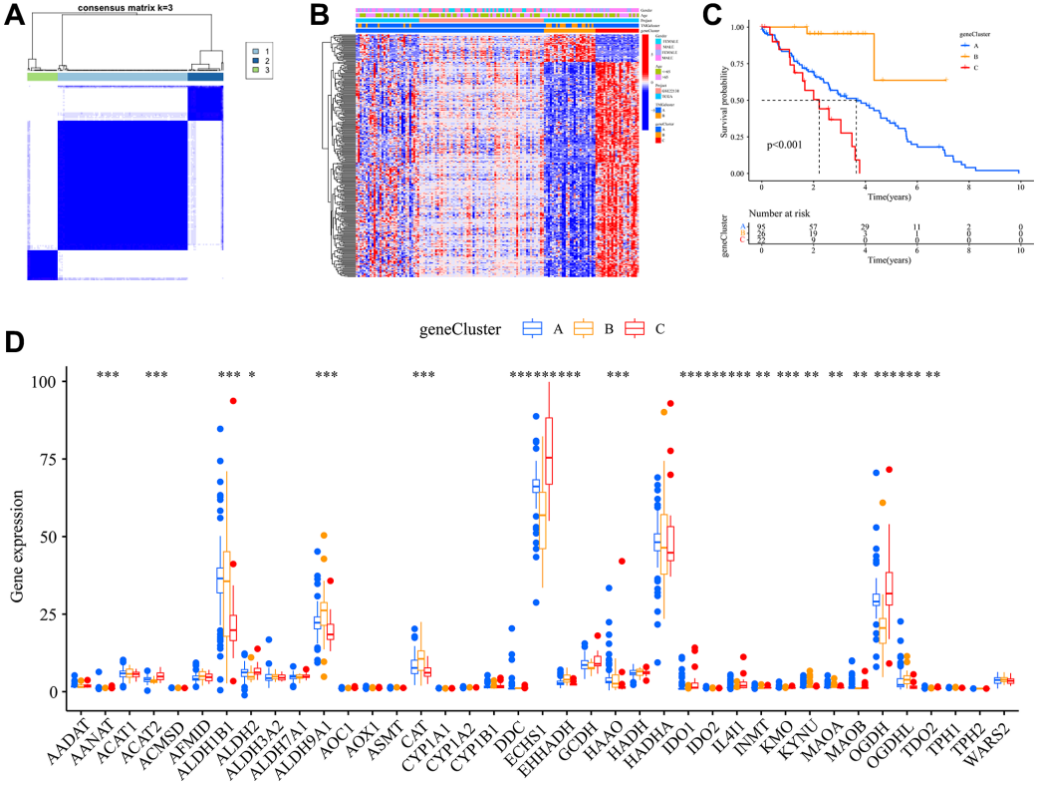
**Construction of gene subtypes.** (**A**) Harmonized matrix heatmap delineating a pair of distinct gene assemblages; (**B**) Disparate expression of TMRGs amid clinical characteristics within the duet of gene subclasses; (**C**) Survival curves for OS of the two gene subtypes; (**D**) Variations in the manifestation of 40 TMRGs between the pair of gene subclasses.

Through comparative analysis of the two Trp subtypes, we identified 994 differentially expressed genes (DEGs) at their intersection. Univariate Cox regression analysis was employed to assess the prognostic significance of these DEGs, leading to the selection of 284 specific genes. Leveraging the insights from the Trp subtypes, we utilized the consensus clustering algorithm to stratify patients into three genomic subtypes based on these 284 prognostic genes, which we termed gene subtypes A, B, and C (Figure 3A). Clinical analysis showed that cluster B had a higher expression of TMRGs compared to cluster C (Figure 3B). Survival analysis underscored the superior survival benefits for individuals in gene cluster A (Figure 3C). A comprehensive review of TMRGs expression levels across the gene subtypes revealed marked differences in genes such as AANAT, ACAT2, and ECHS1, among others (Figure 3D).

### Construction of the prognostic model of TMRGs in UVM

**Figure 4:**
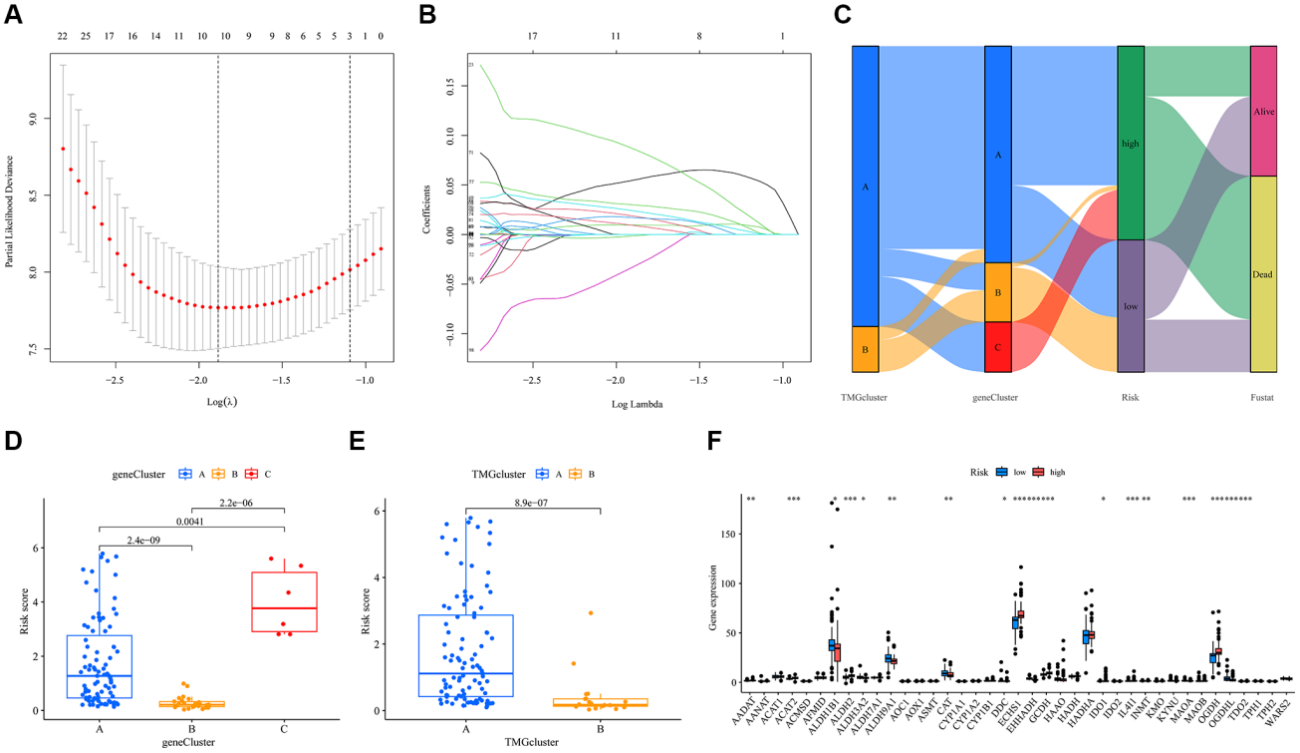
**Construction of the prognostic model for TMRGs in UVM.** (**A**) TMRGs profiles based on LASSO coefficients; (**B**) LASSO coefficient values of the TMGRs in UVM; (**C**) Sankey diagrams of different status, risk score, gene cluster and TMG-associated cluster; (**D**) Risk score in the three gene clusters; (**E**) Risk score in the two TMG-associated cluster. (**F**) Differences in the expression of 40 TMRGs between different risk subgroups.

We subjected UVM patients to LASSO and multivariate Cox regression analyses, identifying five key model genes with their associated coefficients (Figure 4A, 4B). The resulting formula was: risk score = (−0.168 × expression of ATP13A3) + (0.060 × expression of CHST11) + (0.218 × expression of TPST2) + (0.085 × expression of TAPBPL) + (0.045 × expression of MRPL24). Using this risk score, we stratified the patient cohort into high-risk and low-risk groups based on the median signature risk score. A Sankey diagram illustrated the distribution and characteristics of patients, highlighting the majority in the high-risk group who sadly succumbed (Figure 4C). Notably, the risk score distribution varied among TMG and gene clusters: gene cluster C had the highest risk score, while TMRGs cluster A was associated with a higher risk score (Figure 4D, 4E). Moreover, significant differences in TMRGs expression levels, including AADAT, ACAT2, and ECHS1 among others, emerged between the high- risk and low-risk groups (Figure 4F).

**Figure 5:**
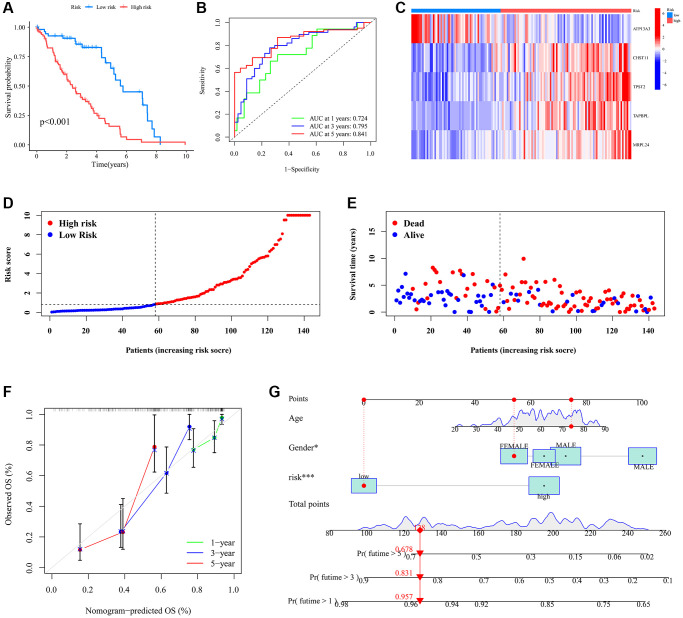
**The prognostic signature’s risk score and its correlation with survival outcomes within the UVM cohort.** (**A**) Survival analysis of the different risk subgroups; (**B**) ROC curves illustrating the predictive accuracy of 1-, 3-, and 5-year survival rates based on varying risk scores; (**C**) Disparate expression of model genes in the different risk subgroups. (**D**) Distribution of the risk score in the different risk subgroups. (**E**) The survival status in the different risk subgroups; (**F**) Nomogram model for individual patient survival probabilities at 1-, 3-, and 5-year intervals for those diagnosed with UVM; (**G**) Calibration curves depicting the nomogram’s accuracy in relation to observed OS at 1-, 3-, and 5-year milestones.

The Kaplan-Meier survival curve demonstrated that the low-risk patient group had a significantly longer OS than the high-risk group (Figure 5A). Time-dependent ROC curves for 1-year, 3-year, and 5-year OS yielded AUCs of 0.724, 0.795, and 0.841, respectively (Figure 5B). A heatmap visualized the expression patterns of the five model genes across different risk score groups (Figure 5C). As risk scores increased, the distribution of survival status showed a rising mortality rate (Figure 5D, 5E). The calibration plot highlighted a strong alignment between observed and predicted outcomes (Figure 5F). Cox analysis results identified both gender and risk score as independent prognostic factors for UVM patients (Figure 5G). Collectively, these results underscore the reliability of the risk score as a prognostic tool for UVM.

### Immune infiltration and drug susceptibility in UVM

**Figure 6:**
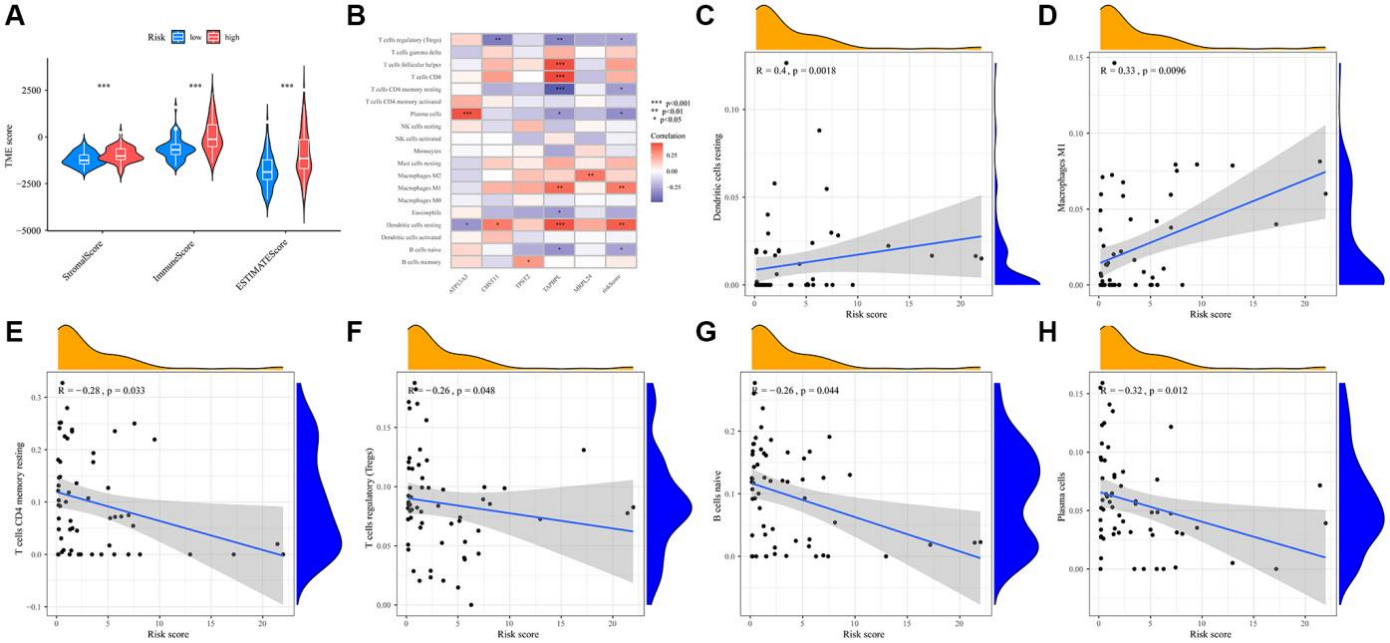
**Prognostic TMRGs intervene in immune infiltration in UVM.** (**A**) Correlations between risk score and immune, stromal and ESTIMATE scores; (**B**) Heatmap of the immune cells, five differential gene and the risk score; (**C**–**H**) Correlation between the risk score and the abundance of immune cells, including (**C**) Dendritic cells resting, (**D**) Macrophages M1, (**E**) T cells CD4 memory resting, (**F**) T cells regulatory (Tregs), (**G**) B cells naive, (**H**) Plasma cells.

Upon a thorough examination of immune scores, we discerned that the high-risk group demonstrated a pronounced increase in their stromal, immune, and ESTIMATE scores (Figure 6A). To further elucidate differences in immune infiltration, we compared the low- and high-risk groups (Figure 6B). Notably, high- risk patients showed a significant presence of certain immune cells, notably resting dendritic cells (Figure 6C) and M1 macrophages (Figure 6D). Conversely, the low-risk group was characterized by a higher abundance of T cells CD4 memory resting (Figure 6E), regulatory T cells (Tregs) (Figure 6F), naive B cells (Figure 6G), and plasma cells (Figure 6H).

**Figure 7:**
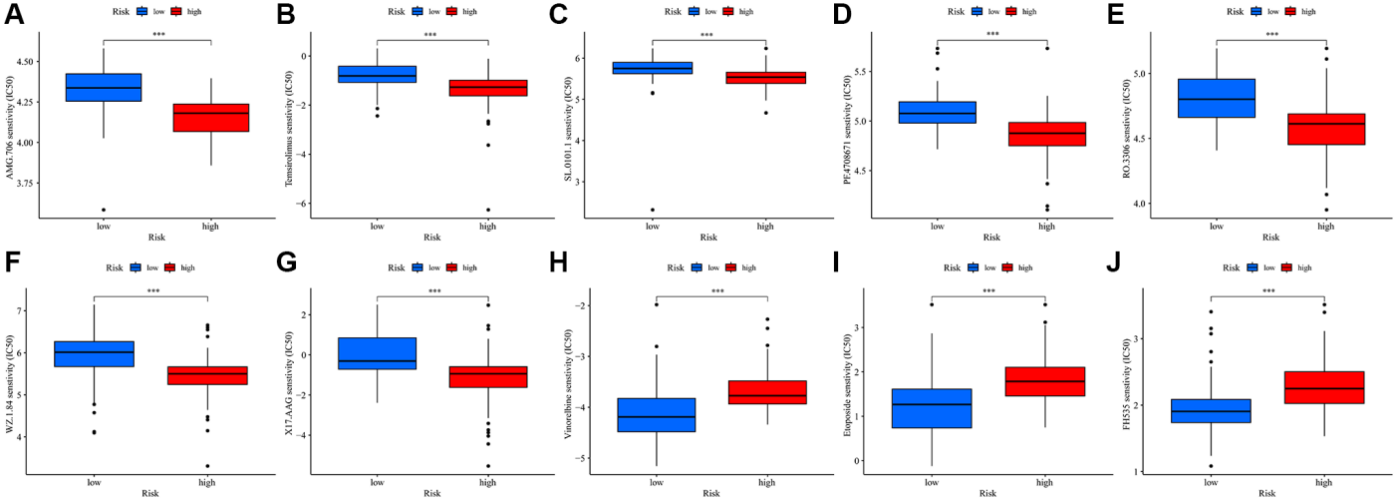
**Drug sensitivity for TMRGs in UVM.** (**A**–**I**) Correlations between the risk score and AMG. 706 (**A**), temsirolimus (**B**), SL.0101.1 (**C**), PF.4708671 (**D**), RO.3306 (**E**), WZ.1.84 (**F**), X17.AAG (**G**), Vinorelbine (**H**), Etoposide (**I**), and FH535 (**J**) sensitivity.

Our study also sought to assess the responsiveness of patients in different risk score groups to common immunotherapeutic drugs. Interestingly, the high-risk group predominantly showed lower IC50 values for several drugs, namely AMG.706, temsirolimus, SL.0101.1, PF.4708671, RO.3306, WZ.1.84, and X17.AAG (Figure 7A–7G). On the other hand, the low-risk group displayed lower IC50 values for vinorelbine, etoposide, and FH535 (Figure 7H–7J). From this comprehensive data analysis, we deduced that high-risk patients tend to have a heightened responsiveness to immunotherapy.

### The effect of Trp on tumorigenicity of EC cell

**Figure 8:**
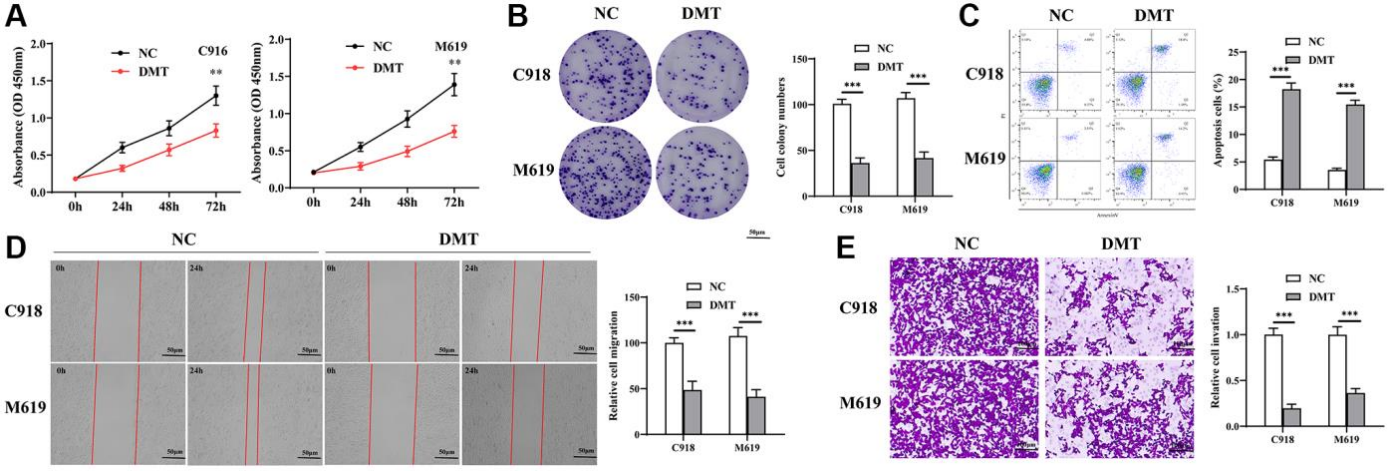
**The effect of TPM on UVM cells.** (**A**, **B**) Cell proliferation detected using CCK-8 assay (**A**) and colony formation assay (**B**); (**C**) Cell apoptosis detected using flow cytometry; (**D**) Cell migration measured using wound healing test; (**E**) Cell invasive ability detected using Transwell assay. Abbreviation: TPM: tryptophan metabolism.

In our efforts to understand the effects of Trp on the malignant behaviors of UVM cells, we utilized DMT as a Trp inhibitor and evaluated its impact on cell proliferation, apoptosis, migration, and invasion. Our results from the CCK-8 assay (Figure 8A) and colony formation assay (Figure 8B) indicated that inhibiting Trp effectively suppressed UVM cell proliferation. Flow cytometry analyses further revealed that Trp inhibition significantly enhanced apoptosis in UVM cells (Figure 8C). Additionally, we noted a marked reduction in cell migration distance upon Trp inhibition (Figure 8D). Moreover, the cells treated with the Trp inhibitor displayed a reduced propensity to invade Matrigel compared to the control cells (Figure 8E). In essence, Trp plays a pivotal role in the tumorigenicity of UVM cells.

## DISCUSSION

In adults, UVM is the most common primary intraocular malignancy, affecting approximately five individuals per million each year [27]. While localized ocular treatments such as brachytherapy, proton beam therapy, and enucleation have been employed, they only partially inhibit tumor growth [28]. Unfortunately, once metastasis occurs, the patient’s life expectancy sharply decreases [29]. If left untreated, a concerning 80–90% of UVM patients will face metastasis [30]. Despite significant advancements in diagnostics and treatments over the past decade, metastasis rates persistently remain around 50%, with the majority of patients succumbing within a year [31]. Identifying prognostic risk factors or molecular markers could facilitate the early removal of emerging metastatic sites and more careful monitoring, potentially improving both the quality and duration of life.

Numerous studies have emphasized the crucial role of TPM in cancer progression [14, 32]. Decreased levels of TPM have been reported in various malignancies, including UVM [33]. Importantly, TPM plays a vital role in T-cell regulation and immune cell infiltration within tumor environments [34]. The expression of IDO1 has been shown to correlate with immune infiltration in multiple types of cancer [35]. Motivated by these findings, we sought to investigate the relationship between Trp, cancer traits, and the immune landscape in UVM. To this end, we delved into analyzing the TRMGs expression patterns in UVM, striving to decipher the intricate associations between these genes, clinicopathological features, and the broader immune context of the disease.

We began by examining the variations in TRMGs expression in UVM patients from the TCGA cohort, recognizing the intricate interplay between gene expression, methylation changes, and their potential influence on patient survival. Our findings highlighted those aberrant expressions of WARS1, KMP, IDO1, OGDHL, HAAO, EHHADH, ALDH9A1, ALDH1B1, and AANAT were significantly associated with reduced survival rates in UVM patients. Elevated methylation levels of ALDH7A1 and EHHADH, as well as decreased methylation of IDO2, MAOB, INMT, and DDC, were found to correlate with unfavorable patient outcomes. Additionally, we identified CNVs across all TRMGs. Based on these observations, we propose that TPH1, IDO2, IDO1, EHHADH, and CAT could be pivotal prognostic markers for UVM patients.

Following a rigorous univariate Cox regression analysis, we identified a specific set of eight TRMGs (specifically, ALDH9A1, CAT, CYP1A1, ECHS1, EHHADH, IL4I1, OGDH, and TPH2). Using these genes, we established two Trp subcategories based on patient data from TCGA and GSE22138 databases.

ALDH9A1, a member of the aldehyde dehydrogenases (ALDH) family, facilitates the conversion of gamma- aminobutyraldehyde (ABAL) to GABA. Notably, Zhang et al. [36] highlighted its association with melanoma development and drug resistance. The CAT gene encodes catalase, an essential antioxidant enzyme defending against oxidative stress. An imbalance in antioxidants has been associated with melanoma progression [37]. Recent studies indicate a heightened expression of CYP1A1 in G0-positive melanoma cells, emphasizing its significance in cancer progression [38]. The ECHS1 gene, crucial for fatty acid beta-oxidation, interacts with STAT3 [39] and has been linked to metastatic melanoma. EHHADH, essential for the peroxisomal beta-oxidation process, has shown increased mRNA expression in some melanoma cell lines [40]. IL4I1, expressed by CD11b+ myeloid cells, modifies the immune landscape in melanoma [41]. In melanoma cells with suppressed SIRT3, OGDH expression was found reduced [42]. Lastly, TPH1, involved in 5-methoxytryptophan synthesis, exhibited increased levels in melanoma cells compared to tumor- infiltrating lymphocytes [43]. Collectively, these genes offer promising avenues for inhibiting UVM malignancy by altering metabolism and immune regulation.

Among the subtypes, subtype A showed a more favorable survival outcome. GO and KEGG enrichment analyses, performed using GSVA, revealed that subtype A was mainly enriched in cancer and metabolic pathways. Furthermore, based on prognosis-related DEGs, three distinct gene subtypes were identified. Of these, subtype A presented a survival advantage over the others, underscoring the prognostic potential of our Trp-associated signature.

Following the Cox analysis, we identified ATP13A3, CHST11, TPST2, TAPBPL, and MRRL24 as significant prognostic markers for UVM in both the TCGA and GSE22138 cohorts, leading to the development of a prognostic model. Notably, the risk score distribution showed variation across both the TMG and gene clusters. Patients categorized in the low- risk group had notably longer overall survival than those in the high-risk group. The accuracy and reliability of this prognostic signature were affirmed through internal validation, using both ROC and calibration chart evaluations. Further analysis from the Cox assessment identified gender and risk score as independent prognostic factors for UVM patients. While some studies found no gender differences in UVM outcomes [44], others have reported increased metastasis rates and shorter survival times for male patients [6]. This aligns with our findings, where male patients predominantly fell into the high-risk category and showed a correlation with reduced UVM survival durations.

Exploring the intricate immune landscapes of UVM, we observed a deep interconnection between TPM and the immunological intricacies of the disease. Patients in the high-risk group exhibited pronounced increases in stromal, immune, and ESTIMATE scores. Our CIBERSORTx analysis revealed a marked infiltration of diverse immune cells, particularly macrophages and T cells, which interact closely with TRMGs. Notably, M1 macrophages displayed a significant association with TRMGs. These M1 macrophages play a critical role in promoting tumor growth and inducing immunosuppressive effects [45]. Our findings suggest that high TRMGs values, indicative of increased tryptophan catabolism and reduced tryptophan availability, may signal an increased infiltration of M1 macrophages, leading to immunosuppression [46]. Tumors have been observed to recruit circulating monocytes and local macrophages, triggering a shift from M1 to M2 macrophages [47]. This transformation results in the formation of tumor-associated macrophages (TAMs), which produce cytokines known to inhibit T-cell activity and increase immunosuppressive proteins [48]. Our analysis revealed a strong negative association between T-cells and TRMGs. The immunosuppressive capabilities of TAMs facilitate immune evasion in UVM, suggesting a poor prognosis for UVM patients with elevated TRMGs.

The field of tumor immunotherapy aims to enhance the body’s defense against tumor evasion while minimizing adverse effects [49]. This therapeutic approach encompasses immune checkpoint inhibitors (ICIs), thymosin, and biologically-derived cells such as dendritic cells and chimeric antigen receptor T cells [50]. Notably, CTLA-4, PD-1, and PD-L1 have gained prominence in clinical research due to their demonstrated safety and efficacy [51]. The application of ICIs against UVM has shown promise. Our findings reveal increased expression of checkpoints in high-risk patients, indicating the potential for improved responsiveness to treatment. These high-risk patients might have a greater sensitivity to ICIs, and drugs like AMG.706, temsirolimus, SL.0101.1, PF.4708671, RO.3306, WZ.1.84, and X17.AAG could offer therapeutic benefits within this vulnerable group.

In our effort to understand the effects of Trp on UVM malignancy *in vitro*, we performed various assays on UVM cell lines C918 and M619. These assays included evaluations of cell viability, apoptosis, migration, and invasiveness. Post-DMT treatment, we observed a decrease in cell viability and a reduction in the migratory and invasive abilities of UVM cells. On the other hand, apoptosis in UVM cells increased. These results highlight the suppressive effect of Trp on UVM cells, emphasizing its importance in UVM progression and hinting at its potential therapeutic value for UVM patients.

The newly developed prognostic model, centered on the expression of five critical TMRGs, presents a transformative approach for clinicians. By distinguishing patients into high-risk and low-risk categories, it enables tailored surveillance strategies, allowing for adaptable frequency of consultations and depth of examinations. This stratification becomes all the more vital, as the Kaplan-Meier survival curves demonstrate a markedly reduced overall survival for the high-risk cohort. A nuanced grasp of the UVM immune landscape, as delineated by the TMRG model, unveils promising therapeutic possibilities. The pronounced prevalence of specific immune cells, such as resting dendritic cells and M1 macrophages in the high-risk faction, compared to their counterparts in the low-risk segment, provides invaluable insights for immunotherapy interventions. Amplifying this perspective is the finding that individuals in the high-risk category display an enhanced susceptibility to select immunotherapeutic drugs. Recognizing the clinical ramifications of TMRGs can steer future investigations towards uncovering correlated genes, interconnected metabolic pathways, or external determinants influencing TMRGs’ expression or function. Periodical assessments of TMRGs expression or tracking their metabolic by-products could serve as a barometer for treatment success or the looming threat of recurrence.

While our study aligns with several others that report similar findings, it’s not without limitations. The main challenges include differences in sequencing methods and data preprocessing across the two cohorts studied. Additionally, as our model was based on existing databases, caution is advised when applying our findings to the broader patient population. Furthermore, the detailed functions and regulations of Trp genes in both *in vitro* and *in vivo* settings warrant deeper investigation. Lastly, the exact mechanism through which TPM affects UVM’s immune environment remains a puzzle that calls for more in-depth research and understanding.

## CONCLUSION

Our findings elucidate the close association between the expression patterns of TrMGs and the clinicopathological and immunological characteristics of UVM. The novel TPM assessment score system exhibits a strong potential to predict the outcomes for individuals diagnosed with UVM. Moreover, a higher risk score—suggesting increased tryptophan catabolism and reduced tryptophan availability—foreshadows enhanced immune infiltration, immunosuppression, and the presence of numerous immunotherapeutic targets. Such insights underscore the potential of using TRMGs to guide immunotherapy approaches in UVM.

## Supplementary Materials

Supplementary File 1

Supplementary File 2

Supplementary File 3

Supplementary File 4

Supplementary File 5
