# Supplementary material for: Tryptophan metabolism-related gene expression patterns: unveiling prognostic insights and immune landscapes in uveal melanoma
Source: Aging (Albany NY). 2023 Oct 13;15(20):11201–16. doi: 10.18632/aging.205122 (PMC10637787; doi:10.18632/aging.205122)
Supplement: Supplementary File 5 [file aging-15-205122-s005.docx]

**Supplementary File 5. The R code script used in this study.**

```

library(survival)

library(survminer)

library(ConsensusClusterPlus)

library(limma)

library(GSEABase)

library(GSVA)

library(pheatmap)

library(reshape2)

library(ggpubr)

library(survival)

library(caret)

library(glmnet)

library(timeROC)

library(ggalluvial)

library(ggplot2)

library(dplyr)

library(regplot)

library(rms)

expFile="GeneExp.txt"

cliFile="time.txt"

data=read.table(expFile, header=T, sep="\t", check.names=F, row.names=1)

data=t(data)

data2=data

rownames(data)=gsub("(.*?)\\_(.*?)", "\\2", rownames(data))

cli=read.table(cliFile, header=T, sep="\t", check.names=F, row.names=1)

sameSample=intersect(row.names(data), row.names(cli))

data=data[sameSample,]

cli=cli[sameSample,]

rt=cbind(cli, data)

sigGenes=c()

for(i in colnames(rt)[3:ncol(rt)]){

cox=coxph(Surv(futime, fustat) ~ rt[,i], data = rt)

coxSummary=summary(cox)

coxP=coxSummary$coefficients[,"Pr(>|z|)"]

if(coxP<0.05){ sigGenes=c(sigGenes,i) }

}

maxK=9

data=t(data2[,sigGenes])

results=ConsensusClusterPlus(data,

maxK=maxK,

reps=50,

pItem=0.8,

pFeature=1,

title=workDir,

clusterAlg="pam",

distance="euclidean",

seed=123456,

plot="png")

sameSample=intersect(row.names(cluster), row.names(cli))

rt=cbind(cli[sameSample,,drop=F], cluster[sameSample,,drop=F])

length=length(levels(factor(rt$TMGcluster)))

diff=survdiff(Surv(futime, fustat) ~ TMGcluster, data = rt)

pValue=1-pchisq(diff$chisq, df=length-1)

if(pValue<0.001){

pValue="p<0.001"

}else{

pValue=paste0("p=",sprintf("%.03f",pValue))

}

fit <- survfit(Surv(futime, fustat) ~ TMGcluster, data = rt)

print(surv_median(fit))

bioCol=c("#0066FF","#FF9900")

bioCol=bioCol[1:length]

surPlot=ggsurvplot(fit,

data=rt,

conf.int=F,

pval=pValue,

pval.size=6,

legend.title="TMGcluster",

legend.labs=levels(factor(rt[,"TMGcluster"])),

legend = c(0.8, 0.8),

font.legend=10,

xlab="Time(years)",

break.time.by = 2,

palette = bioCol,

surv.median.line = "hv",

risk.table=T,

cumevents=F,

risk.table.height=.25)

pdf(file="survival.pdf", width=6.5, height=5.25, onefile=FALSE)

expFile="merge.txt"

clusterFile="TMGcluster.txt"

gmtFile="c2.cp.kegg.symbols.gmt"

rt=read.table(expFile, header=T, sep="\t", check.names=F)

exp=rt[,2:ncol(rt)]

dimnames=list(rownames(exp), colnames(exp))

data=matrix(as.numeric(as.matrix(exp)), nrow=nrow(exp), dimnames=dimnames)

data=avereps(data)

geneSets=getGmt(gmtFile, geneIdType=SymbolIdentifier())

gsvaResult=gsva(data,

geneSets,

min.sz=10,

max.sz=500,

verbose=TRUE,

parallel.sz=1)

gsvaOut=rbind(id=colnames(gsvaResult), gsvaResult)

cluster=read.table(clusterFile, header=T, sep="\t", check.names=F, row.names=1)

gsvaResult=t(gsvaResult)

sameSample=intersect(row.names(gsvaResult), row.names(cluster))

gsvaResult=gsvaResult[sameSample,,drop=F]

cluster=cluster[sameSample,,drop=F]

gsvaCluster=cbind(gsvaResult, cluster)

Project=gsub("(.*?)\\_.*", "\\1", rownames(gsvaCluster))

gsvaCluster=cbind(gsvaCluster, Project)

adj.P.Val.Filter=0.05

allType=as.vector(gsvaCluster$TMGcluster)

comp=combn(levels(factor(allType)), 2)

for(i in 1:ncol(comp)){

treat=gsvaCluster[gsvaCluster$TMGcluster==comp[2,i],]

con=gsvaCluster[gsvaCluster$TMGcluster==comp[1,i],]

data=rbind(con, treat)

Type=as.vector(data$TMGcluster)

ann=data[,c(ncol(data), (ncol(data)-1))]

data=t(data[,-c((ncol(data)-1), ncol(data))])

design=model.matrix(~0+factor(Type))

colnames(design)=levels(factor(Type))

fit=lmFit(data, design)

contrast=paste0(comp[2,i], "-", comp[1,i])

cont.matrix=makeContrasts(contrast, levels=design)

fit2=contrasts.fit(fit, cont.matrix)

fit2=eBayes(fit2)

allDiff=topTable(fit2,adjust='fdr',number=200000)

allDiffOut=rbind(id=colnames(allDiff),allDiff)

diffSig=allDiff[with(allDiff, (abs(logFC)>0.1 & adj.P.Val < adj.P.Val.Filter )), ]

diffSigOut=rbind(id=colnames(diffSig),diffSig)

write.table(diffSigOut, file=paste0(contrast, ".diff.txt"), sep="\t", quote=F, col.names=F)

bioCol=c("#0066FF","#FF9900","#FF0000","#6E568C","#7CC767","#223D6C","#D20A13","#FFD121","#088247","#11AA4D")

ann_colors=list()

m6aCluCol=bioCol[1:length(levels(factor(allType)))]

names(m6aCluCol)=levels(factor(allType))

ann_colors[["TMGcluster"]]=m6aCluCol[c(comp[1,i], comp[2,i])]

termNum=20

diffTermName=as.vector(rownames(diffSig))

diffLength=length(diffTermName)

if(diffLength<termNum){termNum=diffLength}

hmGene=diffTermName[1:termNum]

hmExp=data[hmGene,]

pdf(file=paste0(contrast,".heatmap.pdf"), width=10, height=6)

pheatmap(hmExp,

annotation=ann,

annotation_colors = ann_colors,

color = colorRampPalette(c(rep("blue",2), "white", rep("red",2)))(50),

cluster_cols =F,

show_colnames = F,

gaps_col=as.vector(cumsum(table(Type))),

scale="row",

fontsize = 8,

fontsize_row=6,

fontsize_col=8)

dev.off()

}

gmtFile="c5.go.symbols.gmt"

geneSets=getGmt(gmtFile, geneIdType=SymbolIdentifier())

gsvaResult=gsva(data,

geneSets,

min.sz=10,

max.sz=500,

verbose=TRUE,

parallel.sz=1)

gsvaOut=rbind(id=colnames(gsvaResult), gsvaResult)

gsvaResult=t(gsvaResult)

sameSample=intersect(row.names(gsvaResult), row.names(cluster))

gsvaResult=gsvaResult[sameSample,,drop=F]

cluster=cluster[sameSample,,drop=F]

gsvaCluster=cbind(gsvaResult, cluster)

Project=gsub("(.*?)\\_.*", "\\1", rownames(gsvaCluster))

gsvaCluster=cbind(gsvaCluster, Project)

adj.P.Val.Filter=0.05

allType=as.vector(gsvaCluster$TMGcluster)

comp=combn(levels(factor(allType)), 2)

for(i in 1:ncol(comp)){

treat=gsvaCluster[gsvaCluster$TMGcluster==comp[2,i],]

con=gsvaCluster[gsvaCluster$TMGcluster==comp[1,i],]

data=rbind(con, treat)

Type=as.vector(data$TMGcluster)

ann=data[,c(ncol(data), (ncol(data)-1))]

data=t(data[,-c((ncol(data)-1), ncol(data))])

design=model.matrix(~0+factor(Type))

colnames(design)=levels(factor(Type))

fit=lmFit(data, design)

contrast=paste0(comp[2,i], "-", comp[1,i])

cont.matrix=makeContrasts(contrast, levels=design)

fit2=contrasts.fit(fit, cont.matrix)

fit2=eBayes(fit2)

allDiff=topTable(fit2,adjust='fdr',number=200000)

allDiffOut=rbind(id=colnames(allDiff),allDiff)

diffSig=allDiff[with(allDiff, (abs(logFC)>0.1 & adj.P.Val < adj.P.Val.Filter )), ]

diffSigOut=rbind(id=colnames(diffSig),diffSig)

bioCol=c("#0066FF","#FF9900","#FF0000","#6E568C","#7CC767","#223D6C","#D20A13","#FFD121","#088247","#11AA4D")

ann_colors=list()

m6aCluCol=bioCol[1:length(levels(factor(allType)))]

names(m6aCluCol)=levels(factor(allType))

ann_colors[["TMGcluster"]]=m6aCluCol[c(comp[1,i], comp[2,i])]

termNum=20

diffTermName=as.vector(rownames(diffSig))

diffLength=length(diffTermName)

if(diffLength<termNum){termNum=diffLength}

hmGene=diffTermName[1:termNum]

hmExp=data[hmGene,]

pdf(file=paste0(contrast,".heatmap.pdf"), width=10, height=6)

pheatmap(hmExp,

annotation=ann,

annotation_colors = ann_colors,

color = colorRampPalette(c(rep("blue",2), "white", rep("red",2)))(50),

cluster_cols =F,

show_colnames = F,

gaps_col=as.vector(cumsum(table(Type))),

scale="row",

fontsize = 8,

fontsize_row=6,

fontsize_col=8)

dev.off()

}

expFile="merge.txt"

clusterFile="TMGcluster.txt"

gmtFile="immune.gmt"

rt=read.table(expFile, header=T, sep="\t", check.names=F)

rt=as.matrix(rt)

rownames(rt)=rt[,1]

exp=rt[,2:ncol(rt)]

data=matrix(as.numeric(as.matrix(exp)),nrow=nrow(exp),dimnames=dimnames)

data=avereps(data)

geneSets=getGmt(gmtFile, geneIdType=SymbolIdentifier())

ssgseaScore=gsva(data, geneSets, method='ssgsea', kcdf='Gaussian', abs.ranking=TRUE)

normalize=function(x){

return((x-min(x))/(max(x)-min(x)))}

ssgseaScore=normalize(ssgseaScore)

ssgseaOut=rbind(id=colnames(ssgseaScore), ssgseaScore)

cluster=read.table(clusterFile, header=T, sep="\t", check.names=F, row.names=1)

ssgseaScore=t(ssgseaScore)

sameSample=intersect(row.names(ssgseaScore), row.names(cluster))

ssgseaScore=ssgseaScore[sameSample,,drop=F]

cluster=cluster[sameSample,,drop=F]

scoreCluster=cbind(ssgseaScore, cluster)

data=melt(scoreCluster, id.vars=c("TMGcluster"))

colnames(data)=c("TMGcluster", "Immune", "Fraction")

bioCol=c("#0066FF","#FF9900","#FF0000","#6E568C","#7CC767","#223D6C","#D20A13","#FFD121","#088247","#11AA4D")

bioCol=bioCol[1:length(levels(factor(data[,"TMGcluster"])))]

p=ggboxplot(data, x="Immune", y="Fraction", color="TMGcluster",

ylab="Immune infiltration",

xlab="",

legend.title="TMGcluster",

palette=bioCol)

p=p+rotate_x_text(50)

pdf(file="boxplot.pdf", width=8, height=6.5)

p+stat_compare_means(aes(group=TMGcluster),symnum.args=list(cutpoints = c(0, 0.001, 0.01, 0.05, 1), symbols = c("***", "**", "*", "")),label = "p.signif")

dev.off()

expFile="interGeneExp.txt"

cliFile="time.txt"

data=read.table(expFile, header=T, sep="\t", check.names=F, row.names=1)

cli=read.table(cliFile, header=T, sep="\t", check.names=F, row.names=1)

sameSample=intersect(row.names(data), row.names(cli))

data=data[sameSample,]

cli=cli[sameSample,]

rt=cbind(cli,data)

sigGenes=c()

for(i in colnames(rt)[3:ncol(rt)]){

cox=coxph(Surv(futime, fustat) ~ rt[,i], data = rt)

coxSummary=summary(cox)

coxP=coxSummary$coefficients[,"Pr(>|z|)"]

if(coxP<0.001){ sigGenes=c(sigGenes,i) }

}

outTab=data2[,sigGenes]

outTab=cbind(id=row.names(outTab), outTab)

outTab=rt[,c("futime","fustat",sigGenes)]

outTab=cbind(id=row.names(outTab), outTab)

maxK=9

data=t(data2[,sigGenes])

results=ConsensusClusterPlus(data,

maxK=maxK,

reps=50,

pItem=0.8,

pFeature=1,

title=workDir,

clusterAlg="pam",

distance="euclidean",

seed=123456,

plot="png")

exp=read.table(expFile, header=T, sep="\t", check.names=F, row.names=1)

tmgClu=read.table(tmgCluFile, header=T, sep="\t", check.names=F, row.names=1)

geneClu=read.table(geneCluFile, header=T, sep="\t", check.names=F, row.names=1)

sameSample=intersect(row.names(exp), row.names(tmgClu))

exp=exp[sameSample,,drop=F]

expData=cbind(exp, geneCluster=geneClu[sameSample,], TMGcluster=tmgClu[sameSample,])

Project=gsub("(.*?)\\_.*", "\\1", rownames(expData))

rownames(expData)=gsub("(.*?)\\_(.*?)", "\\2", rownames(expData))

expData=cbind(expData, Project)

cli=read.table(cliFile, header=T, sep="\t", check.names=F, row.names=1)

cli[,"Age"]=ifelse(cli[,"Age"]=="unknow", "unknow", ifelse(cli[,"Age"]>65,">65","<=65"))

sameSample=intersect(row.names(expData), row.names(cli))

expData=expData[sameSample,,drop=F]

cli=cli[sameSample,,drop=F]

data=cbind(expData, cli)

data=data[order(data$geneCluster),]

Type=data[,((ncol(data)-2-ncol(cli)):ncol(data))]

data=t(data[,1:(ncol(expData)-3)])

bioCol=c("#0066FF","#FF9900","#FF0000","#6E568C","#7CC767","#223D6C","#D20A13","#FFD121","#088247","#11AA4D")

ann_colors=list()

TMGcol=bioCol[1:length(levels(factor(Type$TMGcluster)))]

names(TMGcol)=levels(factor(Type$TMGcluster))

ann_colors[["TMGcluster"]]=TMGcol

GENEcol=bioCol[1:length(levels(factor(Type$geneCluster)))]

names(GENEcol)=levels(factor(Type$geneCluster))

ann_colors[["geneCluster"]]=GENEcol

pdf("heatmap.pdf", height=6, width=8)

pheatmap(data,

annotation=Type,

annotation_colors = ann_colors,

color = colorRampPalette(c(rep("blue",5), "white", rep("red",5)))(50),

cluster_cols =F,

cluster_rows =T,

scale="row",

show_colnames=F,

show_rownames=F,

fontsize=6,

fontsize_row=6,

fontsize_col=6)

dev.off()

clusterFile="geneCluster.txt"

cliFile="time.txt"

cluster=read.table(clusterFile, header=T, sep="\t", check.names=F, row.names=1)

rownames(cluster)=gsub("(.*?)\\_(.*?)", "\\2", rownames(cluster))

cli=read.table(cliFile, header=T, sep="\t", check.names=F, row.names=1)

colnames(cli)=c("futime", "fustat")

cli$futime=cli$futime/365

sameSample=intersect(row.names(cluster), row.names(cli))

rt=cbind(cli[sameSample,,drop=F], cluster[sameSample,,drop=F])

length=length(levels(factor(rt$geneCluster)))

diff=survdiff(Surv(futime, fustat) ~ geneCluster, data = rt)

pValue=1-pchisq(diff$chisq, df=length-1)

if(pValue<0.001){

pValue="p<0.001"

}else{

pValue=paste0("p=",sprintf("%.03f",pValue))

}

fit <- survfit(Surv(futime, fustat) ~ geneCluster, data = rt)

print(surv_median(fit))

bioCol=c("#0066FF","#FF9900","#FF0000")

bioCol=bioCol[1:length(levels(factor(rt[,"geneCluster"])))]

surPlot=ggsurvplot(fit,

data=rt,

conf.int=F,

pval=pValue,

pval.size=6,

legend.title="geneCluster",

legend.labs=levels(factor(rt[,"geneCluster"])),

legend = c(0.8, 0.8),

font.legend=10,

xlab="Time(years)",

break.time.by = 2,

palette = bioCol,

surv.median.line = "hv",

risk.table=T,

cumevents=F,

risk.table.height=.25)

pdf(file="survival.pdf", width=7, height=5.5, onefile=FALSE)

dev.off()

expFile="tmgGeneExp.txt"

geneCluFile="geneCluster.txt"

rt=read.table(expFile, header=T, sep="\t", check.names=F)

rt=as.matrix(rt)

rownames(rt)=rt[,1]

exp=rt[,2:ncol(rt)]

dimnames=list(rownames(exp),colnames(exp))

data=matrix(as.numeric(as.matrix(exp)),nrow=nrow(exp),dimnames=dimnames)

data=avereps(data)

data=t(data)

geneClu=read.table(geneCluFile, header=T, sep="\t", check.names=F, row.names=1)

sameSample=intersect(row.names(data), row.names(geneClu))

expClu=cbind(data[sameSample,,drop=F], geneClu[sameSample,,drop=F])

data=melt(expClu, id.vars=c("geneCluster"))

colnames(data)=c("geneCluster", "Gene", "Expression")

data=data[-c(861,883,887,2787),]

bioCol=c("#0066FF","#FF9900","#FF0000","#6E568C","#7CC767","#223D6C","#D20A13","#FFD121","#088247","#11AA4D")

bioCol=bioCol[1:length(levels(factor(data[,"geneCluster"])))]

p=ggboxplot(data, x="Gene", y="Expression", color = "geneCluster",

xlab="",

ylab="Gene expression",

legend.title="geneCluster",

palette = bioCol,

width=0.8,

size=0.3

)

p=p+rotate_x_text(45)

p1=p+stat_compare_means(aes(group=geneCluster),

symnum.args=list(cutpoints = c(0, 0.001, 0.01, 0.05, 1), symbols = c("***", "**", "*", " ")),

label = "p.signif")

pdf(file="boxplot.pdf", width=10, height=5)

dev.off()

rt=read.table("uniSigExpTime.txt", header=T, sep="\t", check.names=F, row.names=1)

rt$futime[rt$futime<=1]=1

rt$futime=rt$futime/365

for(i in 1:n){

inTrain<-createDataPartition(y=rt[,2], p=0.5, list=F)

train<-rt[inTrain,]

test<-rt[-inTrain,]

trainOut=cbind(id=row.names(train),train)

testOut=cbind(id=row.names(test),test)

x=as.matrix(train[,c(3:ncol(train))])

y=data.matrix(Surv(train$futime,train$fustat))

fit <- glmnet(x, y, family = "cox", maxit = 1000)

cvfit <- cv.glmnet(x, y, family="cox", maxit = 1000)

coef <- coef(fit, s = cvfit$lambda.min)

index <- which(coef != 0)

actCoef <- coef[index]

lassoGene=row.names(coef)[index]

lassoSigExp=train[,c("futime", "fustat", lassoGene)]

lassoSigExpOut=cbind(id=row.names(lassoSigExp), lassoSigExp)

geneCoef=cbind(Gene=lassoGene, Coef=actCoef)

if(nrow(geneCoef)<2){next}

multiCox <- coxph(Surv(futime, fustat) ~ ., data = lassoSigExp)

multiCox=step(multiCox,direction = "both")

multiCoxSum=summary(multiCox)

outMultiTab=data.frame()

outMultiTab=cbind(

coef=multiCoxSum$coefficients[,"coef"],

HR=multiCoxSum$conf.int[,"exp(coef)"],

HR.95L=multiCoxSum$conf.int[,"lower .95"],

HR.95H=multiCoxSum$conf.int[,"upper .95"],

pvalue=multiCoxSum$coefficients[,"Pr(>|z|)"])

outMultiTab=cbind(id=row.names(outMultiTab),outMultiTab)

outMultiTab=outMultiTab[,1:2]

riskScore=predict(multiCox,type="risk",newdata=train)

coxGene=rownames(multiCoxSum$coefficients)

coxGene=gsub("`","",coxGene)

outCol=c("futime","fustat",coxGene)

medianTrainRisk=median(riskScore)

risk=as.vector(ifelse(riskScore>medianTrainRisk,"high","low"))

trainRiskOut=cbind(id=rownames(cbind(train[,outCol],riskScore,risk)),cbind(train[,outCol],riskScore,risk))

riskScoreTest=predict(multiCox,type="risk",newdata=test)

riskTest=as.vector(ifelse(riskScoreTest>medianTrainRisk,"high","low"))

testRiskOut=cbind(id=rownames(cbind(test[,outCol],riskScoreTest,riskTest)),cbind(test[,outCol],riskScore=riskScoreTest,risk=riskTest))

diff=survdiff(Surv(futime, fustat) ~risk,data = train)

pValue=1-pchisq(diff$chisq, df=1)

diffTest=survdiff(Surv(futime, fustat) ~riskTest,data = test)

pValueTest=1-pchisq(diffTest$chisq, df=1)

predictTime=3

roc=timeROC(T=train$futime, delta=train$fustat,

marker=riskScore, cause=1,

times=c(predictTime), ROC=TRUE)

rocTest=timeROC(T=test$futime, delta=test$fustat,

marker=riskScoreTest, cause=1,

times=c(predictTime), ROC=TRUE)

if((pValue<0.01) & (roc$AUC[2]>0.65) & (pValueTest<0.05) & (rocTest$AUC[2]>0.6)){

write.table(trainOut,file="data.train.txt",sep="\t",quote=F,row.names=F)

write.table(testOut,file="data.test.txt",sep="\t",quote=F,row.names=F)

write.table(lassoSigExpOut,file="lasso.SigExp.txt",sep="\t",row.names=F,quote=F)

pdf("lasso.lambda.pdf")

plot(fit, xvar = "lambda", label = TRUE)

dev.off()

pdf("lasso.cvfit.pdf")

plot(cvfit)

abline(v=log(c(cvfit$lambda.min,cvfit$lambda.1se)), lty="dashed")

dev.off()

}

}

tmgCluFile="TMGcluster.txt"

geneCluFile="geneCluster.txt"

riskFile="risk.all.txt"

tmgClu=read.table(tmgCluFile, header=T, sep="\t", check.names=F, row.names=1)

geneClu=read.table(geneCluFile, header=T, sep="\t", check.names=F, row.names=1)

risk=read.table(riskFile, header=T, sep="\t", check.names=F, row.names=1)

twoCluster=cbind(tmgClu, geneClu)

rownames(twoCluster)=gsub("(.*?)\\_(.*?)", "\\2", rownames(twoCluster))

sameSample=intersect(row.names(twoCluster), row.names(risk))

rt=cbind(risk[sameSample,,drop=F], twoCluster[sameSample,,drop=F])

rt=rt[,c("TMGcluster", "geneCluster", "risk", "fustat")]

colnames(rt)=c("TMGcluster", "geneCluster", "Risk", "Fustat")

rt[,"Fustat"]=ifelse(rt[,"Fustat"]==0, "Alive", "Dead")

corLodes=to_lodes_form(rt, axes = 1:ncol(rt), id = "Cohort")

pdf(file="ggalluvial.pdf", width=6, height=5.5)

mycol=rep(c("#0066FF","#FF9900","#FF0000","#029149","#6E568C","#E0367A","#D8D155","#223D6C","#D20A13","#431A3D","#91612D","#FFD121","#088247","#11AA4D","#58CDD9","#7A142C","#5D90BA","#64495D","#7CC767"),15)

ggplot(corLodes, aes(x = x, stratum = stratum, alluvium = Cohort,fill = stratum, label = stratum)) +

scale_x_discrete(expand = c(0, 0)) +

geom_flow(width = 2/10,aes.flow = "forward") +

geom_stratum(alpha = .9,width = 2/10) +

scale_fill_manual(values = mycol) +

geom_text(stat = "stratum", size = 3,color="black") +

xlab("") + ylab("") + theme_bw() +

theme(axis.line = element_blank(),axis.ticks = element_blank(),axis.text.y = element_blank()) +

theme(panel.grid =element_blank()) +

theme(panel.border = element_blank()) +

ggtitle("") + guides(fill = FALSE)

dev.off()

riskFile$riskScore[score$riskScore>quantile(score$riskScore,0.99)]=quantile(score$riskScore,0.99)

twoCluster=cbind(tmgCluFile, geneCluFile)

rownames(twoCluster)=gsub("(.*?)\\_(.*?)", "\\2", rownames(twoCluster))

sameSample=intersect(row.names(twoCluster), row.names(score))

data=cbind(score[sameSample,,drop=F], twoCluster[sameSample,,drop=F])

data=data[order(data$riskScore),]

data=data[-c(120:143),]

data$TMGcluster=factor(data$TMGcluster, levels=levels(factor(data$TMGcluster)))

group=levels(factor(data$TMGcluster))

comp=combn(group, 2)

my_comparisons=list()

for(i in 1:ncol(comp)){my_comparisons[[i]]<-comp[,i]}

bioCol=c("#0066FF","#FF9900")

bioCol=bioCol[1:length(levels(factor(data$TMGcluster)))]

boxplot=ggboxplot(data, x="TMGcluster", y="riskScore", color="TMGcluster",

xlab="TMGcluster",

ylab="Risk score",

legend.title="TMGcluster",

palette=bioCol,

add = "jitter")+

stat_compare_means(comparisons = my_comparisons)

pdf(file="TMGcluster.pdf", width=5, height=4.5)

print(boxplot)

dev.off()

data$geneCluster=factor(data$geneCluster, levels=levels(factor(data$geneCluster)))

group=levels(factor(data$geneCluster))

comp=combn(group, 2)

my_comparisons=list()

for(i in 1:ncol(comp)){my_comparisons[[i]]<-comp[,i]}

bioCol=c("#0066FF","#FF9900","#FF0000")

bioCol=bioCol[1:length(levels(factor(data$geneCluster)))]

boxplot=ggboxplot(data, x="geneCluster", y="riskScore", color="geneCluster",

xlab="geneCluster",

ylab="Risk score",

legend.title="geneCluster",

palette=bioCol,

add = "jitter")+

stat_compare_means(comparisons = my_comparisons)

pdf(file="geneCluster.pdf", width=5, height=4.5)

print(boxplot)

dev.off()

expFile="TMGGeneExp.txt"

riskFile="risk.all.txt"

rt=read.table(expFile, header=T, sep="\t", check.names=F)

rt=as.matrix(rt)

rownames(rt)=rt[,1]

exp=rt[,2:ncol(rt)]

dimnames=list(rownames(exp),colnames(exp))

data=matrix(as.numeric(as.matrix(exp)),nrow=nrow(exp),dimnames=dimnames)

data=avereps(data)

data=t(data)

rownames(data)=gsub("(.*?)\\_(.*?)", "\\2", rownames(data))

risk=read.table(riskFile, sep="\t", header=T, check.names=F, row.names=1)

sameSample=intersect(row.names(data),row.names(risk))

rt1=cbind(data[sameSample,,drop=F],risk[sameSample,"risk",drop=F])

rt1=melt(rt1,id.vars=c("risk"))

colnames(rt1)=c("risk","Gene","Expression")

group=levels(factor(rt1$risk))

rt1$risk=factor(rt1$risk, levels=c("low","high"))

comp=combn(group,2)

my_comparisons=list()

for(j in 1:ncol(comp)){my_comparisons[[j]]<-comp[,j]}

boxplot=ggboxplot(rt1, x="Gene", y="Expression", fill="risk",

xlab="",

ylab="Gene expression",

legend.title="Risk",

width=0.8,

palette = c("#0088FF", "#FF5555") )+

rotate_x_text(50)+

stat_compare_means(aes(group=risk),

method="wilcox.test",

symnum.args=list(cutpoints=c(0, 0.001, 0.01, 0.05, 1), symbols=c("***", "**", "*", "")), label="p.signif")

pdf(file="genediff.pdf", width=10, height=5)

print(boxplot)

dev.off()

#定义生存分析函数

bioSurvival=function(inputFile=null, outFile=null){

#读取输入文件

rt=read.table(inputFile, header=T, sep="\t", check.names=F)

rt$risk=factor(rt$risk, levels=c("low", "high"))

#比较高低风险组生存差异，得到生存差异的p值

diff=survdiff(Surv(futime, fustat) ~risk,data = rt)

pValue=1-pchisq(diff$chisq,df=1)

if(pValue<0.001){

pValue="p<0.001"

}else{

pValue=paste0("p=",sprintf("%.03f",pValue))

}

fit <- survfit(Surv(futime, fustat) ~ risk, data = rt)

surPlot=ggsurvplot(fit,

data=rt,

conf.int=F,

pval=pValue,

pval.size=6,

legend.title="Risk",

legend.labs=c("Low risk", "High risk"),

xlab="Time(years)",

break.time.by = 2,

palette=c("#0088FF", "#FF5555"),

risk.table=F,

risk.table.title="",

risk.table.col = "strata",

risk.table.height=.25)

pdf(file=outFile, width=5, height=4.5, onefile=FALSE)

print(surPlot)

dev.off()

}

bioSurvival(inputFile="risk.all.txt", outFile="surv.all.pdf")

bioROC=function(inputFile=null, rocFile=null){

rt=read.table(inputFile, header=T, sep="\t", check.names=F)

ROC_rt=timeROC(T=rt$futime,delta=rt$fustat,

marker=rt$riskScore,cause=1,

weighting='aalen',

times=c(1,3,5),ROC=TRUE)

pdf(file=rocFile, width=5, height=5)

plot(ROC_rt,time=1,col='green',title=FALSE,lwd=2)

plot(ROC_rt,time=3,col='blue',add=TRUE,title=FALSE,lwd=2)

plot(ROC_rt,time=5,col='red',add=TRUE,title=FALSE,lwd=2)

legend('bottomright',

c(paste0('AUC at 1 years: ',sprintf("%.03f",ROC_rt$AUC[1])),

paste0('AUC at 3 years: ',sprintf("%.03f",ROC_rt$AUC[2])),

paste0('AUC at 5 years: ',sprintf("%.03f",ROC_rt$AUC[3]))),

col=c("green",'blue','red'),lwd=2,bty = 'n')

dev.off()

}

bioROC(inputFile="risk.all.txt", rocFile="ROC.all.pdf")

bioRiskPlot=function(inputFile=null, project=null){

rt=read.table(inputFile, header=T, sep="\t", check.names=F, row.names=1)

rt=rt[order(rt$riskScore),]

riskClass=rt[,"risk"]

lowLength=length(riskClass[riskClass=="low"])

highLength=length(riskClass[riskClass=="high"])

lowMax=max(rt$riskScore[riskClass=="low"])

line=rt[,"riskScore"]

line[line>10]=10

pdf(file=paste0(project, ".riskScore.pdf"), width=7, height=4)

plot(line, type="p", pch=20,

xlab="Patients (increasing risk socre)",

ylab="Risk score",

col=c(rep("blue",lowLength),rep("red",highLength)) )

abline(h=lowMax,v=lowLength,lty=2)

legend("topleft", c("High risk","Low Risk"),bty="n",pch=19,col=c("red","blue"),cex=1.2)

dev.off()

color=as.vector(rt$fustat)

color[color==1]="red"

color[color==0]="blue"

pdf(file=paste0(project, ".survStat.pdf"), width=7, height=4)

plot(rt$futime, pch=19,ylim=c(0,18),

xlab="Patients (increasing risk socre)",

ylab="Survival time (years)",

col=color)

legend("topleft", c("Dead","Alive"),bty="n",pch=19,col=c("red","blue"),cex=1.2)

abline(v=lowLength,lty=2)

dev.off()

ann_colors=list()

bioCol=c("#0088FF", "#FF5555")

names(bioCol)=c("low", "high")

ann_colors[["Risk"]]=bioCol

rt1=rt[c(3:(ncol(rt)-2))]

rt1=t(rt1)

annotation=data.frame(Risk=rt[,ncol(rt)])

rownames(annotation)=rownames(rt)

pdf(file=paste0(project, ".heatmap.pdf"), width=7, height=4)

pheatmap(rt1,

annotation=annotation,

annotation_colors = ann_colors,

cluster_cols = FALSE,

cluster_rows = FALSE,

show_colnames = F,

scale="row",

color = colorRampPalette(c(rep("blue",3.5), "white", rep("red",3.5)))(50),

fontsize_col=7,

fontsize=7,

fontsize_row=8)

dev.off()

}

bioRiskPlot(inputFile="risk.all.txt", project="all")

riskFile="risk.all.txt"

cliFile="clinical.txt"

risk=read.table(riskFile, header=T, sep="\t", check.names=F, row.names=1)

cli=read.table(cliFile, header=T, sep="\t", check.names=F, row.names=1)

cli=cli[apply(cli,1,function(x)any(is.na(match('unknow',x)))),,drop=F]

cli$Age=as.numeric(cli$Age)

samSample=intersect(row.names(risk), row.names(cli))

risk1=risk[samSample,,drop=F]

cli=cli[samSample,,drop=F]

rt=cbind(risk1[,c("futime", "fustat", "risk")], cli)

res.cox=coxph(Surv(futime, fustat) ~ . , data = rt)

nom1=regplot(res.cox,

plots = c("density", "boxes"),

clickable=F,

title="",

points=TRUE,

droplines=TRUE,

observation=rt[2,],

rank="sd",

failtime = c(1,3,5),

prfail = F)

nomoRisk=predict(res.cox, data=rt, type="risk")

rt=cbind(risk1, Nomogram=nomoRisk)

outTab=rbind(ID=colnames(rt), rt)

write.table(outTab, file="nomoRisk.txt", sep="\t", col.names=F, quote=F)

pdf(file="calibration.pdf", width=5, height=5)

f <- cph(Surv(futime, fustat) ~ Nomogram, x=T, y=T, surv=T, data=rt, time.inc=1)

cal <- calibrate(f, cmethod="KM", method="boot", u=1, m=(nrow(rt)/3), B=1000)

plot(cal, xlim=c(0,1), ylim=c(0,1),

xlab="Nomogram-predicted OS (%)", ylab="Observed OS (%)", lwd=1.5, col="green", sub=F)

f <- cph(Surv(futime, fustat) ~ Nomogram, x=T, y=T, surv=T, data=rt, time.inc=3)

cal <- calibrate(f, cmethod="KM", method="boot", u=3, m=(nrow(rt)/3), B=1000)

plot(cal, xlim=c(0,1), ylim=c(0,1), xlab="", ylab="", lwd=1.5, col="blue", sub=F, add=T)

f <- cph(Surv(futime, fustat) ~ Nomogram, x=T, y=T, surv=T, data=rt, time.inc=5)

cal <- calibrate(f, cmethod="KM", method="boot", u=5, m=(nrow(rt)/3), B=1000)

plot(cal, xlim=c(0,1), ylim=c(0,1), xlab="", ylab="", lwd=1.5, col="red", sub=F, add=T)

legend('bottomright', c('1-year', '3-year', '5-year'),

col=c("green","blue","red"), lwd=1.5, bty = 'n')

dev.off()

#' CIBERSORT R script v1.03

#' Note: Signature matrix construction is not currently available; use java version for full functionality.

#' Author: Aaron M. Newman, Stanford University (amnewman@stanford.edu)

#' Requirements:

#' R v3.0 or later. (dependencies below might not work properly with earlier versions)

#' install.packages('e1071')

#' install.pacakges('parallel')

#' install.packages('preprocessCore')

#' if preprocessCore is not available in the repositories you have selected, run the following:

#' source("http://bioconductor.org/biocLite.R")

#' biocLite("preprocessCore")

#' Windows users using the R GUI may need to Run as Administrator to install or update packages.

#' This script uses 3 parallel processes. Since Windows does not support forking, this script will run

#' single-threaded in Windows.

#'

#' Usage:

#' Navigate to directory containing R script

#'

#' In R:

#' source('CIBERSORT.R')

#' results <- CIBERSORT('sig_matrix_file.txt','mixture_file.txt', perm, QN)

#'

#' Options:

#' i) perm = No. permutations; set to >=100 to calculate p-values (default = 0)

#' ii) QN = Quantile normalization of input mixture (default = TRUE)

#'

#' Input: signature matrix and mixture file, formatted as specified at http://cibersort.stanford.edu/tutorial.php

#' Output: matrix object containing all results and tabular data written to disk 'CIBERSORT-Results.txt'

#' License: http://cibersort.stanford.edu/CIBERSORT_License.txt

#' Core algorithm

#' @param X cell-specific gene expression

#' @param y mixed expression per sample

#' @export

CoreAlg <- function(X, y){

#try different values of nu

svn_itor <- 3

res <- function(i){

if(i==1){nus <- 0.25}

if(i==2){nus <- 0.5}

if(i==3){nus <- 0.75}

model<-svm(X,y,type="nu-regression",kernel="linear",nu=nus,scale=F)

model

}

if(Sys.info()['sysname'] == 'Windows') out <- mclapply(1:svn_itor, res, mc.cores=1) else

out <- mclapply(1:svn_itor, res, mc.cores=svn_itor)

nusvm <- rep(0,svn_itor)

corrv <- rep(0,svn_itor)

#do cibersort

t <- 1

while(t <= svn_itor) {

weights = t(out[[t]]$coefs) %*% out[[t]]$SV

weights[which(weights<0)]<-0

w<-weights/sum(weights)

u <- sweep(X,MARGIN=2,w,'*')

k <- apply(u, 1, sum)

nusvm[t] <- sqrt((mean((k - y)^2)))

corrv[t] <- cor(k, y)

t <- t + 1

}

#pick best model

rmses <- nusvm

mn <- which.min(rmses)

model <- out[[mn]]

#get and normalize coefficients

q <- t(model$coefs) %*% model$SV

q[which(q<0)]<-0

w <- (q/sum(q))

mix_rmse <- rmses[mn]

mix_r <- corrv[mn]

newList <- list("w" = w, "mix_rmse" = mix_rmse, "mix_r" = mix_r)

}

#' do permutations

#' @param perm Number of permutations

#' @param X cell-specific gene expression

#' @param y mixed expression per sample

#' @export

doPerm <- function(perm, X, Y){

itor <- 1

Ylist <- as.list(data.matrix(Y))

dist <- matrix()

while(itor <= perm){

#print(itor)

#random mixture

yr <- as.numeric(Ylist[sample(length(Ylist),dim(X)[1])])

#standardize mixture

yr <- (yr - mean(yr)) / sd(yr)

#run CIBERSORT core algorithm

result <- CoreAlg(X, yr)

mix_r <- result$mix_r

#store correlation

if(itor == 1) {dist <- mix_r}

else {dist <- rbind(dist, mix_r)}

itor <- itor + 1

}

newList <- list("dist" = dist)

}

CIBERSORT <- function(sig_matrix, mixture_file, perm=0, QN=TRUE){

library(e1071)

library(parallel)

library(preprocessCore)

X <- read.table(sig_matrix,header=T,sep="\t",row.names=1,check.names=F)

Y <- read.table(mixture_file, header=T, sep="\t", row.names=1,check.names=F)

X <- data.matrix(X)

Y <- data.matrix(Y)

X <- X[order(rownames(X)),]

Y <- Y[order(rownames(Y)),]

P <- perm #number of permutations

if(max(Y) < 50) {Y <- 2^Y}

if(QN == TRUE){

tmpc <- colnames(Y)

tmpr <- rownames(Y)

Y <- normalize.quantiles(Y)

colnames(Y) <- tmpc

rownames(Y) <- tmpr

}

Xgns <- row.names(X)

Ygns <- row.names(Y)

YintX <- Ygns %in% Xgns

Y <- Y[YintX,]

XintY <- Xgns %in% row.names(Y)

X <- X[XintY,]

X <- (X - mean(X)) / sd(as.vector(X))

if(P > 0) {nulldist <- sort(doPerm(P, X, Y)$dist)}

header <- c('Mixture',colnames(X),"P-value","Correlation","RMSE")

output <- matrix()

itor <- 1

mixtures <- dim(Y)[2]

pval <- 9999

while(itor <= mixtures){

y <- Y[,itor]

y <- (y - mean(y)) / sd(y)

result <- CoreAlg(X, y)

w <- result$w

mix_r <- result$mix_r

mix_rmse <- result$mix_rmse

if(P > 0) {pval <- 1 - (which.min(abs(nulldist - mix_r)) / length(nulldist))}

out <- c(colnames(Y)[itor],w,pval,mix_r,mix_rmse)

if(itor == 1) {output <- out}

else {output <- rbind(output, out)}

itor <- itor + 1

}

write.table(rbind(header,output), file="CIBERSORT-Results.txt", sep="\t", row.names=F, col.names=F, quote=F)

obj <- rbind(header,output)

obj <- obj[,-1]

obj <- obj[-1,]

obj <- matrix(as.numeric(unlist(obj)),nrow=nrow(obj))

rownames(obj) <- colnames(Y)

colnames(obj) <- c(colnames(X),"P-value","Correlation","RMSE")

obj

}

inputFile="merge.txt"

rt=read.table(inputFile, header=T, sep="\t", check.names=F)

rt=as.matrix(rt)

rownames(rt)=rt[,1]

exp=rt[,2:ncol(rt)]

dimnames=list(rownames(exp),colnames(exp))

data=matrix(as.numeric(as.matrix(exp)),nrow=nrow(exp),dimnames=dimnames)

data=avereps(data)

data=data[rowMeans(data)>0,]

out=rbind(ID=colnames(data),data)

write.table(out,file="uniq.symbol.txt",sep="\t",quote=F,col.names=F)

source("cuproOmics42.CIBERSORT.R")

results=CIBERSORT("ref.txt", "uniq.symbol.txt", perm=1000)

riskFile="risk.all.txt"

TMEfile="TMEscores.txt"

Risk=read.table(riskFile, header=T, sep="\t", check.names=F, row.names=1)

Risk$risk=factor(Risk$risk, levels=c("low","high"))

score=read.table(TMEfile, header=T, sep="\t", check.names=F, row.names=1)

score=score[,1:3]

rownames(score)=gsub("(.*?)\\_(.*?)", "\\2", rownames(score))

score=score[row.names(Risk),,drop=F]

rt=cbind(Risk[,"risk",drop=F], score)

data=melt(rt, id.vars=c("risk"))

colnames(data)=c("Risk", "scoreType", "Score")

p=ggviolin(data, x="scoreType", y="Score", fill = "Risk",

xlab="",

ylab="TME score",

legend.title="Risk",

add = "boxplot", add.params = list(color="white"),

palette = c("#0088FF", "#FF5555"), width=1)

p=p+rotate_x_text(45)

p1=p+stat_compare_means(aes(group=Risk),

method="wilcox.test",

symnum.args=list(cutpoints = c(0, 0.001, 0.01, 0.05, 1), symbols = c("***", "**", "*", " ")),

label = "p.signif")

pdf(file="vioplot.pdf", width=6, height=5)

print(p1)

dev.off()

```
